# Visual Cells Remember Earlier Applied Target: Plasticity of Orientation Selectivity

**DOI:** 10.1371/journal.pone.0003689

**Published:** 2008-11-10

**Authors:** Narcis Ghisovan, Abdellatif Nemri, Svetlana Shumikhina, Stephane Molotchnikoff

**Affiliations:** Department of Biological Sciences, University of Montreal, Montreal, Canada; Centre de Recherches su la Cognition Animale–Centre National de la Recherche Scientifique and Université Paul Sabatier, France

## Abstract

**Background:**

A canonical proposition states that, in mature brain, neurons responsive to sensory stimuli are tuned to specific properties installed shortly after birth. It is amply demonstrated that that neurons in adult visual cortex of cats are orientation-selective that is they respond with the highest firing rates to preferred oriented stimuli.

**Methodology/Principal Findings:**

In anesthetized cats, prepared in a conventional fashion for single cell recordings, the present investigation shows that presenting a stimulus uninterruptedly at a non-preferred orientation for twelve minutes induces changes in orientation preference. Across all conditions orientation tuning curves were investigated using a trial by trial method. Contrary to what has been previously reported with shorter adaptation duration, twelve minutes of adaptation induces mostly attractive shifts, i.e. toward the adapter. After a recovery period allowing neurons to restore their original orientation tuning curves, we carried out a second adaptation which produced three major results: (1) more frequent attractive shifts, (2) an increase of their magnitude, and (3) an additional enhancement of responses at the new or acquired preferred orientation. Additionally, we also show that the direction of shifts depends on the duration of the adaptation: shorter adaptation in most cases produces repulsive shifts, whereas adaptation exceeding nine minutes results in attractive shifts, in the same unit. Consequently, shifts in preferred orientation depend on the duration of adaptation.

**Conclusion/Significance:**

The supplementary response improvements indicate that neurons in area 17 keep a memory trace of the previous stimulus properties, thereby upgrading cellular performance. It also highlights the dynamic nature of basic neuronal properties in adult cortex since repeated adaptations modified both the orientation tuning selectivity and the response strength to the preferred orientation. These enhanced neuronal responses suggest that the range of neuronal plasticity available to the visual system is broader than anticipated.

## Introduction

Visual history is well known to affect perception in adult brain. At the neuronal level, repeated or prolonged exposure to a stimulus (adaptation) is classically known to reduce the neurons' responsiveness to the same stimulus. This effect can last from a few seconds to minutes. While adaptation is associated with perceptual errors such as visual illusions, it often correlates with improved stimulus discrimination and a broadening of the perceptual range [1]–[6]. Neurons from visual cortices discharge specifically for luminance variations occurring within their receptive field. In addition, visual neurons display response tuning for image features such as contrast, orientation, motion direction and speed [7], [8]. Orientation selectivity for instance is an emergent property of primary visual cortex (area V1) neurons in felines and primates. This tuning property does not need visual experience and was considered unchangeable after birth [8]–[10]. However, several authors reported that in the adult visual cortex of monkeys and cats it is possible to modify preferred stimuli such as orientation and direction selectivity of targets that optimally excite neurons by a non-preferred adapting stimulus [11], [12]. Two simultaneous effects on the cell's tuning curves were reported: depression for the preferred stimulus, and response enhancement for stimuli away from the adapter. Hence, the tuning curves shift away from the adapting stimulus, in an apparent sliding movement called repulsive shift. Quite less frequently attractive shifts [6], [13]–[17] were reported; in this case after short-term adaptation neurons discharge at their maximal response rate toward the adapter. This adaptation-induced plasticity changed our views on the mechanisms underlying adaptation from simple synaptic fatigue to complex network interactions. In the present investigation we additionally asked whether stimulation history influences responses following a second adaptation.

In line with the above observations new notions have emerged regarding the reorganization of the mature brain and memory processes that appear to incorporate brain plasticity. The evaluation of a memory mechanism may be achieved by assessing performance improvement. At the single cell level, improvement can only be gauged by measuring neuronal responses after several successive “trainings” such as long-term adaptation. The anticipated improvement might be expected from three cellular behaviours. First, the cell responses could become stronger at the new preferred orientation following the second adaptation. Second, the shift in orientation preference could increase from the first to the second adaptation, thus reducing the gap between the adapter and the newly acquired optimal orientation. This is particularly interesting when the first adaptation induced a repulsive shift or even no change in preferred orientation after a first test. Then the second adaptation may compel cells to reverse their shifts to attractive ones i.e. toward the adapter. Third the response enhancement could occur more rapidly by reducing the duration of adaptation if repeated after recovery of tuning properties. Thus, the cell would require a shorter adaptation time to exhibit an increase of responses toward the new preferred orientation.

In this study, we report a form of neuronal plasticity of cortical responses induced by prolonged presentation of particular non-preferred oriented stimulus covering the cells' receptive fields. Several minutes of continuous stimulation produced mostly attractive shifts thus strengthening cells' firing rate toward the adapting stimulus. The same protocol applied a second time elevates the proportion and the amplitude of attractive shifts. Furthermore, and most importantly, the response at the new preferred orientation is significantly improved. This improved performance suggests that the range of neuronal plasticity available to the visual system is broader than anticipated.

## Results

**Figure 1:**
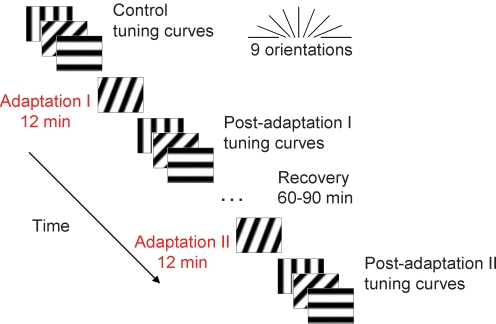
Schematic representation of the experimental adaptation protocol. Responses to sine-wave drifting grating in 9 different orientations, covering multi-units receptive fields, were measured for 25 trials of 4.1 s presented in random order. Adaptation I: orientation tuning curves were plotted prior to and after a 12 min of continuously adaptation to a non-preferred stimulus (22.5°–67.5° off the preferred orientation). Following a recovery period of 60–90 min, orientation tuning curves were replotted. Adaptation II: the same adapting protocol was applied a second time on receptive fields and tuning curves were once again plotted. In additional experiments the duration of adaptation was increased in steps of 3, 6, 9 and 12 min.

To test the effects of repeated adaptations on orientation selectivity, responses of neurons sorted out from multi-units recordings in the primary visual cortex of anesthetized adult cats were studied. In order to determine precisely the plasticity of orientation tuning in our cell population (n = 70 neurons), curve fits were generated using the von Mises Function (see method). Fits accounted for more than 89% of the variance in the data across conditions. The sequence of stimuli presentation is shown in figure 1.

### Adaptation-induced plasticity of orientation tuning

**Figure 2:**
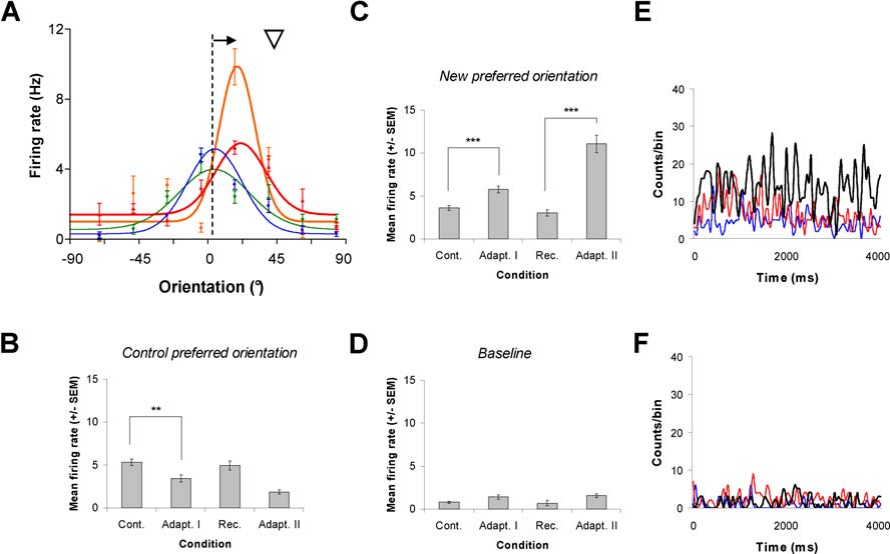
Typical example of shift in orientation preference and response modulations. A: The first 12 min adaptation displaces the preferred orientation of the cell by 15.5° toward the adapting stimulus. The head arrow indicates the non-preferred adapting stimulus. Following a recovery period of 60 min, the cell recovered its control preferred orientation at 9.0°. Adaptation II produces an identical attractive shift of 15.1°. B, C and D: Histograms shows the response modulations at the control preferred orientation, the new preferred orientation after adaptations and the baseline level (θ = 90°), respectively. At the control preferred orientation, the mean firing rate of cell decrease after adaptation I (*t*-test, p<0.001) and returned to control level in 60 min. In parallel, the mean firing rate increase by 27% at the new preferred orientation (*t*-test, p<0.0001). Following recovery, the firing rate further increases: 48% in comparison to adaptation I (*t*-test, p<0.0001). Baseline level remains unchanged across conditions. E and F: Peri-stimulus time histograms (PSTH) are illustrated for the neuron responding to orientations in C and D, respectively. Blue curves; control condition, red curves; adaptation I, black curves; adaptation II.

A typical example of an attractive shift of 15.5° is displayed in figure 2A (averaged tuning curves). In Fig. 2B, C and D mean response modulation of the cell is illustrated through recordings. First, the firing rate at the control optimal orientation (9.1°) dropped by 35% after the first adaptation and by 65% following the second adaptation (paired sample two-tailed *t*-test, p<0.001 and p<0.00001, respectively, Fig. 2B). In parallel, the neurons' response was noticeably increased for the new preferred orientation from 3.6 Hz±0.27 Hz to 5.7 Hz±0.37 Hz (paired sample two-tailed *t*-test, p<0.0001, Fig. 2C). After recovery, a second adaptation of twelve minutes also resulted in an attractive shift of 15.1°. Furthermore, we recorded an additional strengthening (∼50%) of responses at the new preferred orientation (paired sample two-tailed *t*-test, p<0.0001; Fig. 2C). Interestingly, the firing rate at the baseline level (−67.5°≤θ≥67.5°) remained unchanged across conditions (Fig. 2D). Hence the response modulation was constrained around the original and new preferred orientations since the baseline firing rate failed to change (paired sample two-tailed *t*-test, p>0.1; Fig. 2D). The PSTHs illustrate the cumulative response modulations between the control and post-adaptations recordings (see Fig. 2E and 2F). In this example, the dual cellular response modulation leads to a displacement of the peak of the orientation tuning curve in the direction of the adapter. Indeed, attractive and repulsive shifts are often the result of dual modifications of the evoked responses; cells' discharges declined in response to their original preferred orientations whereas responses to the newly acquired optimal orientations are enhanced.

**Figure 3:**
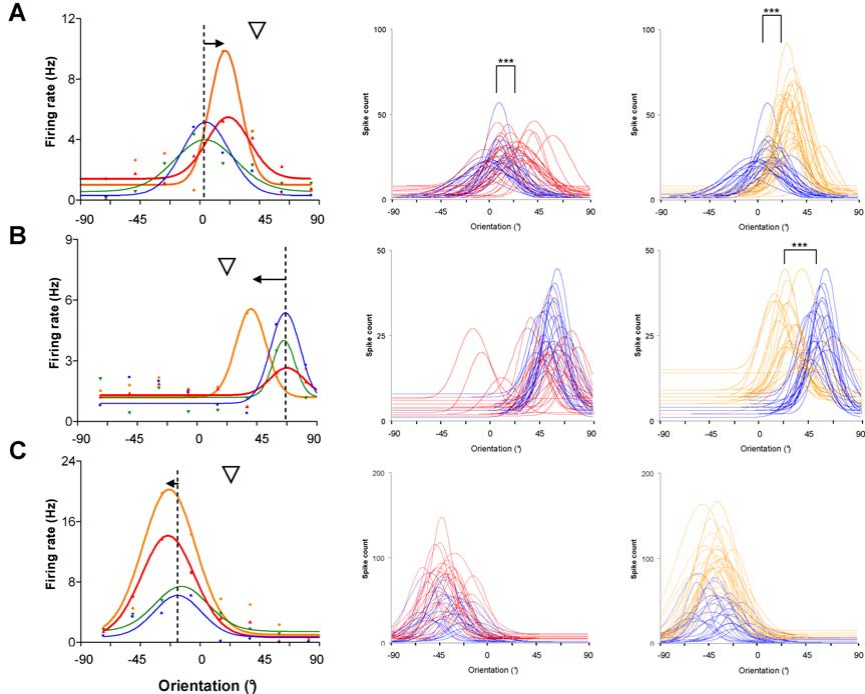
Examples of orientation tuning shifts and response improvements following repeated adaptations. Left column: orientation tuning curves showing averaged responses (pooled data). Middle and right columns: orientation tuning curves changes on trial by trial basis (n = 25 presentations) after adaptation I and II, respectively. A: The first adaptation induced a significant attractive shift of 15.5° (example used in fig. 1, red curves, *t*-test, p<0.0001). After complete recovery, adaptation II produces once again a significant attractive shift. Comparing to the first adaptation, a strong increase of the response is observed at the new preferred stimulus (amber curves, *t*-test, p<0.0001). B: In this example, the cell fails changing its preferred orientation following adaptation I; only response depression is observed (red curves). Adaptation II produces a significant shift of 28.0° (amber curves, *t*-test, p<0.0001). C: Cell displays only weak repulsive shifts of 4.0° after adaptations but its response increase by 27% at the new preferred orientation (compare red to amber curves, *t*-test, p<0.001).

Figure 3 shows three examples of shifts in orientation preference and improvement of evoked responses following adaptations. The middle and the right columns illustrate orientation tuning curves on trial by trial basis (n = 25, see methods). The cell in Fig. 3A displays identical significant attractive shifts following the first and the second adaptation (15.5° and 15.1°; paired sample two-tailed *t*-test, p<0.0001). However, the cell's firing rate strongly increased for the new preferred orientation after a second presentation of the adapter (paired sample two-tailed *t*-test, p<0.0001; same cell as in Fig. 2). In Fig. 3B, the neuron did not shift its preferred orientation after the first adaptation but exhibited a strong significant attractive shift of 28.0° after the second adaptation (paired sample two-tailed *t*-test, p<0.0001). Interestingly, the cell “learned” as the new or acquired optimal orientation approached the adapter stimulus. Because of the difference between the adapting orientation and the original preferred one (Δ≥45.0°), the cell could not reach the adapter. This result was expected from previous reports [18]–[20]. Moreover, this cell shows that the decline of responses by −57% at the control optimal orientation following the first adaptation and the increase at the new preferred orientation (+74%) revealed only after the second adaptation may be dissociated processes. The decrease of discharges in response to the original preferred orientation is not necessarily accompanied by the appearance of a new optimal orientation. Cell in Fig. 3C shows only weak repulsive shifts (4°) but its firing rate increased by 27% at the new preferred orientation following the second adaptation (compare red and amber curves; paired sample two-tailed *t*-test, p<0.001). In all cases, recoveries of control preferred orientation were observed within 60–90 min. In all three cases firing rates outside the range of the response modulation did not significantly change. This suggests that the adaptation modulates cellular activity within a narrow range of orientations, roughly constrained around the initial and the new acquired orientations. Indeed, a statistical survey of our entire population shows that the baseline firing rate remained constant through all experimental steps. Clearly, the modulation of discharges was not induced by a global variation of the firing rate. The stimuli blocking presentations (n = 25 trials) did not modify the cells' optimal firing rates during a recording set. The magnitude of preferred responses varied randomly from a presentation to another rather than progressively decreasing over trials. In our study, shifts in tuning curves were accompanied most of the time by a significant increase of firing rate to stimulus in the direction of the adapter. This neuronal behaviour cannot simply be attributed to neuronal fatigue at the cells' preferred orientation.

**Figure 4:**
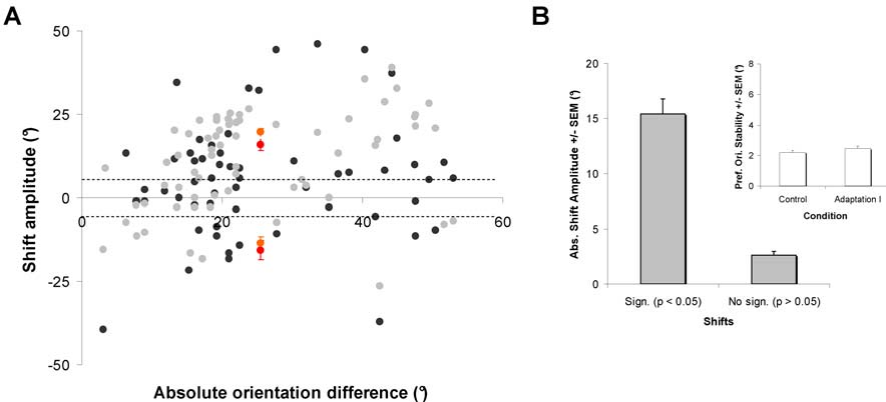
Adaptation-induced plasticity of orientation tuning in a population of 69 neurons. A: Scatter plot showing the amplitude of shifts in preferred orientation after adaptation as a function of the absolute difference between the control preferred orientation and the adapting orientation. Black dots represent shifts orientation preference following adaptation I and gray dots following adaptation II. Positive values designate attractive shifts and negative values designate repulsive shifts. The majority of cells 80% (55/69) displayed significant shifts in orientation preference. Dashed lines represent the significance level. Adaptation I induced mostly significant attractive shifts 74% (41/55) and 26% of significant repulsive shifts (14/55). Overall, the mean attractive shift is 15.7°±1.8°, while the average repulsive shifts is 15.6°±2.9° (red dots; errors bars are SEM). Adaptation II induced more attractive shifts 84% (46/55) than repulsive ones 16% (11/55). The magnitude of the attractive shifts significantly increased to 19.5°±1.3° (Mann Whitney test p<0.01) whereas the mean repulsive shits slightly diminished to 13.6°±2.0° (amber dots; errors bars are SEM). B: Neurons displaying significant (*t*-test, p<0.05) and non-significant (*t*-test, p>0.05) changes in orientation preference are compared regardless of shifts direction (shifts pooled). The mean shift amplitude of orientation is 15.4°+/−1.3° whereas non-significant shifts average 2.6°+/−0.4°. Insert: The jitter in preferred orientations is unchanged following adaptation, prior to adaptation: 2.2°+/−0.02°; following adaptation: 2.4°+/−0.02° I (errors bars are SEM). The histograms suggest that the peak orientation is almost invariant.

Figure 4A shows the shift amplitude on a cell-by-cell basis in relation to the absolute orientation difference between cells' preferred orientation and the adapting stimulus. In our sample, nearly every cell (98%) displayed shifts in preferred orientation (67/69). The majority of shifts (80%) were significant (see below). Following the first adaptation, the attractive shifts were more frequent than repulsive ones (black dots; 74% and 26% respectively). The mean attractive shift was 15.7°±1.8°, while the average repulsive shifts were 15.6°±2.9° (red dots). Since stimuli presented are spaced by 22.5°, curve fits of orientation tuning were purposely generated to determine more precisely the orientation preference. However, there is no significant difference in shift magnitude between fitted tuning curves and raw data [Raw data; Attractive shifts = 14.3°+/−2.1°; Repulsive shifts = 12.8°+/−3.3°]. In addition, the relationships between shift magnitude and significance levels (p values of *t*-test, subset of n = 54) was examined. Neurons displaying attractive and repulsive shifts during adaptation were pooled together to assess only the significance of orientation tuning shifts (Fig. 4B). Shifts in preferred orientation larger than 5° were all statistically significant (paired sample two-tailed *t*-test, *p*<0.05). The mean amplitude of significant shifts reaches 15.4°±1.4° whereas non-significant changes averaged 2.6°±0.4°. The stability of the preferred orientation value across trials was also measured (Fig. 4B; insert). Even though neurons shift their preferred orientation following 12 min of adaptation, the jitter in preferred orientation is rather small and remains unchanged before and after adaptation (control; 2.2°±0.2°, adaptation I; 2.4°±0.2°; paired sample two-tailed, *t*-test p>0.1).

The majority of cells 66% (46/69) recovered their control preferred orientation within 60–90 min (Δ≤5° within control value). The second adaptation increased the occurrence of attractive shifts by 10% while the proportion of repulsive shifts declined (gray dots; 84% and 16%, respectively). Hence, more than the half (12/21) of cells exhibiting initially repulsive shifts reversed the direction of their shifts toward the adaptor. However, most cells (72%) displayed shifts in the same direction as for the first adaptation (50/69). The magnitude of the attractive shifts significantly increased to 19.5°±1.3° (Mann Whitney test p<0.01) whereas the mean repulsive shits slightly diminished to 13.6°±2.0° (Mann Whitney test p>0.1). Both adaptations have no effect on orientation tuning strength; OSIs computed from raw data are largely unchanged after shifts in preferred orientation (control: 0.72±0.02; adaptation I: 0.69±0.02; adaptation II: 0.73±0.02, paired sample two-tailed *t*-test, p>0.05).

### Effects of adaptation duration

**Figure 5:**
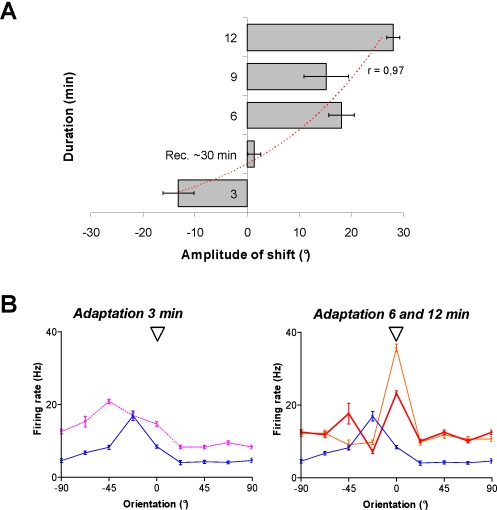
Influence of the adaptation duration on orientation tuning changes. The head arrow indicates the adapting oriented-stimulus (0°). A: Cells were adapted for 3, 6, 9 and 12 min to non-preferred oriented-stimulus (±22.5°–45.0° off the control optimal orientation). After 3 min, cells show repulsive shifts 88% (14/16) averaging 13.2°±3.0°. Following a recovery period of 30 min, cells were again adapted to the same stimulus for 6, 9 and 12 min. Cells displayed almost exclusively 96% (23/24) attractive shifts averaging 18.1°±2.4° and 15.2°±4.2°, for 6 and 9 min, respectively. Twelve minutes of adaptation produced larger attractive shifts of 28.3°±1.2°. B: Example of a cell displaying a repulsive shift of 22.5° after 3 min of adaptation (left column, dashed purple curve). Six minutes of continuous adaptation produces a significant increase of response at the adapting orientation 0° (*t*-test, p<0.0001) but the firing rate still remains high at the preferred orientation −45.0° induced by the previous 3 min adaptation. Twelve minutes of adaptation induces a clear attractive shift of 22.5°.

In contrast to previous reports, our study indicates that attractive shifts are more frequent than repulsive shifts. Consequently we sought to examine if the duration of adaptation may be a contributing factor to this higher occurrence. In additional cells, we increased the duration of our adaptation protocol with a non-preferred stimulus in steps from 3 to 12 min. Successive adaptations of 3, 6, 9 and 12 min were performed on the same cells. Figure 5A shows the average magnitude of shifts in orientation preference (negative values: repulsive shifts; positive values: attractive shifts). After three minutes of adaptation, almost all neurons (14/16) showed repulsive shifts averaging 13.2°±3.0° (two cells exhibited small insignificant shifts in the adapter direction). After 30 min of recovery the cells recovered their preferred orientation. From different set of experiments, cells were successively adapted for 6 and/or 9 min. This resulted almost exclusively in attractive shifts averaging 18.1°±2.4° and 15.2°±4.2° (11/11 cells and 12/13 cells, respectively). Finally, the longest duration of adaptation used in the present study, i.e. 12 min, produced larger attractive shifts (28.3°±1.2°). To summarize repulsive shifts occurred for almost every cell when the adapter is applied for shorter time and shifts were reversed when the duration of adaptation lengthened over six minutes. Tuning curves of the cell in Fig. 5B illustrate a typical example of this shift reversal as the adaptation time increases from three to twelve minutes. After three minutes of adaptation, the cell displayed a net repulsive shift of 22.5°. The firing rate increased by more than 60% at the new preferred orientation (−45.0°; paired sample two-tailed *t*-test, p<0.0001) but remained unchanged at the control preferred one. After 6 min of adaptation, the orientation tuning curve exhibited the following properties (1) the response to the initial optimal orientation has considerably declined by 58% (2) two peaks are revealed by post-adaptation tuning curve. The first peak at −45° recalled the repulsive effect previously observed and the second peak emerged at 0° coinciding with the adapter. Thus, suggesting that the cell was at an intermediate stage oscillating between repulsive and attractive shifts. In this example, raw tuning curves are purposely shown instead of curve fits to illustrate this intermediate stage in relation to the adaptation duration. After twelve minutes of adaptation the neuron displayed a clear attractive shift of 22.5° and its firing rate strongly increased from 8.4 Hz±0.4 Hz to 35.6 Hz±1.1 Hz (paired sample two-tailed *t*-test, p<0.0001).

### Neuronal responses are improved by repeated adaptations

**Figure 6:**
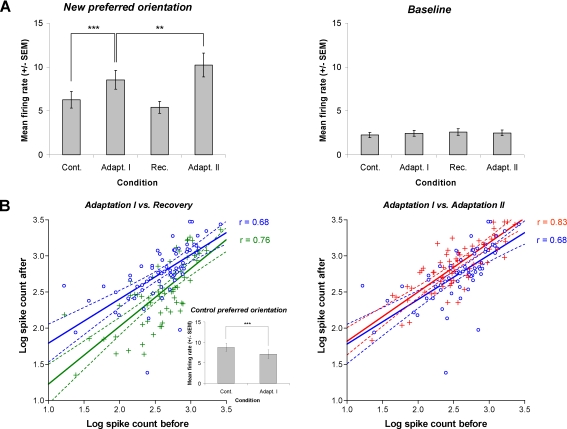
Response modulation across conditions. A: Histograms show the mean firing rate for 2 particular orientations; the new preferred acquired orientation following adaptations and the baseline level (flank orientations). Adaptation I increases the firing rate at the new preferred orientation (*t*-test, p<0.0001). In parallel, the mean response decrease at the control preferred orientation (see insert histogram in B, *t*-test, p<0.0001). The firing returns to its control values within 60–90 min (*t*-test, p>0.1). Adaptation II enhances further the firing rate of 20% over the first one (*t*-test, p<0.001). Baseline remains unchanged across conditions (*t*-test, p>0.1). B: Scatter plots showing *on a cell by cell basis the* modulation of responses at the new preferred stimulus. Data of spike counts were transformed into logarithmic values. Left column: the cells' responses increase after adaptation I (blue circles, r = 0.68) and returned near to control values (green crosses, r = 0.76). Right column: following adaptation II, the cells' responses increase comparing to the first one (red crosses, r = 0.83). Dashed lines indicate the 95% confidence intervals. Correlation coefficients are different between adaptation I and II (Z_D_ = 2.57, p<0.005).

Regardless of the adaptation effect on the orientation preference (attractive vs. repulsive), the modulation of firing rate are measured for three orientations of interest: (1) the new preferred orientation (2) the control preferred orientation (3) the baseline level corresponding to flank orientations. Fig. 6A shows the mean firing rate modulation of the cell population (n = 69) across conditions. The first adaptation significantly increases responses at the new preferred orientation by 27% from 6.2 Hz±0.9 Hz to 8.5 Hz±1.1 Hz (paired sample two-tailed *t*-test, p<0.0001). At the same time, the mean firing rate at the control preferred orientation declined by 20% from 8.8 Hz±1.0 Hz to 7.1 Hz±1.0 Hz (insert histogram from Fig. 6B, paired sample two-tailed *t*-test, p<0.0001). After recovery, the firing rate returned to control values for the new preferred orientation (paired sample two-tailed *t*-test, p>0.1). The second adaptation of 12 min strongly increased the mean firing rate for the new acquired preferred orientation. The average responses nearly doubled from the recovery level reaching 10.3 Hz±1.4 Hz (paired sample two-tailed *t*-test, p<0.001). As mentioned above, the firing rate of the baseline level remained constant across conditions (paired sample two-tailed *t*-test, p>0.1). In addition, spontaneous activity also remains unchanged trough all recording sessions. These above results may not be attributed to occasional surges of spontaneous activity because response modulations were constrained around the preferred orientations. Additionally, the dual effect of response rates reported here at the control preferred orientation (decrease) and at the newly acquired optimal orientation (increase) could not be obtained from a global increase of spontaneous activity.

Figure 6B illustrates on a cell-by-cell basis the magnitude of response modulation specifically at the new preferred orientation induced by both adaptations. In the left scatter plot, data points in control condition were plotted against data obtained after adaptation I (blue circles) and following the recovery (green crosses). Each data point represents spike count responses for individual neurons (n = 69). Clearly, the adaptation enhances cells' firing rate for the new preferred orientation. This enhancement is underlined by the best fitting linear regression (blue line) which is above the equality line. The evoked firing returned to its control level following recovery. Indeed, the trend line (green line) is near to the equality line indicating that cells recovered their control firing rate and thus their control tuning. In the right scatter plot, we compare the response modulation produced by the first adaptation (blue circles) with the second adaptation (red crosses). It shows that the response magnitude originating from the second adaptation lies above the values obtained after the first adaptation thus indicating a strengthening of the cells' discharge. Moreover, the comparison of the data points distribution between the first and the second adaptation is statistically different (r = 0.68, r = 0.83, Z_D_ = 2.57, p<0.005). After a second adaptation data point lie closer to the trend line suggesting that responses become more reliable after repeated adaptations.

## Discussion

We have demonstrated another expression of the plastic nature of mature visual cortex. Previous studies have disclosed the ability of cortical neurons to change their preferred properties following repetitive presentations of non preferred stimuli. Most animals' studies and psychophysical experiments reported that visual adaptation leads to a loss of sensitivity toward the adapter stimulus and resulting in apparent repulsive shifts of tuning properties [4], [11], [21]–[24]. Our data show instead that an uninterrupted long term adaptation produces an increase of spiking rate in response toward the adapter and thus revealed attractive shifts. What is more, we showed that a second adaptation carried out after a period of recovery from the first adaptation further strengthens the responses to the adapter.

### Methodological considerations

It may be argued that since the grating is presented on a relatively dark background its application may result in an increase of screen luminance. Hence, the present results could be attributed to luminance adaptation. However failure to observe similar effects in the LGN is a strong argument that this is not the case. Shou et al. [25] studied LGN responses following prolonged exposure to drifting gratings. Their investigation reports that after grating adaptation responses diminished in most cells. Facilitation was a rare occurrence and no tuning shift was observed. Therefore, the effects that we describe are unlikely to be attributed to contrast adaptation which results in a decline of cortical responses [12], [25]–[28]. Moreover the increased firing rate following uninterrupted application of non-optimal orientation suggests that it is doubtful that neuronal fatigue is involved in the adaptation mechanism. The possibility has been raised that attention increases the effective strength of an attended stimulus thereby increasing the firing rate to that particular stimulus. However, since the animals were anaesthetized the described effects may not be due to variations of attention level. Early reports have also shown that changes in orientation preference are not occurring in the thalamic lateral geniculate nucleus [4], [5], [17], [23], [25]–[28].

Also, it is unlikely that response increases reported in present experiments may be ascribed to a sudden and random increase of spontaneous activity. It has been suggested that the profile of orientation tuning curves varies in relation to cell's discharge variability, and stimulus dimensions covering the periphery of the receptive field [29]–[32]. Alternatively, others have reported that orientation selectivity is invariant with stimulus contrast [33]. Indeed as shown in results section, although response magnitude may vary from trial to trial the jitter of the optimal orientation remain small (fig 5B). Moreover, response modulations following adaptation are constrained roughly around the adapter and the initial preferred orientations while the flanked orientations (all orientations being applied randomly) are unchanged through the recording sessions. Lastly, evoked discharges to the adapter are augmented while in parallel responses to the original preferred orientation are weakened. All the above arguments suggest that it is very unlikely that the specific response modulations are caused by spontaneous surge of global excitability.

### Relationship to earlier studies

The results of the present investigation stand in contrast to previous studies. Dragoi et al. [11] reported that in the majority of cells shifts in a repulsive direction relative to the axis of orientation of the adapter. These differences in the adaptation outcome (attractive vs. repulsive shifts) are rather intriguing. The difference in the adaptation protocol may explain the divergence. In earlier studies the adaptation duration was relatively short (2–3-min) and resulting mostly in repulsive shifts, while in the present study a longer adaptation caused attractive shifts. Dragoi et al. [11] also studied the time course of adaptation and recovery. In their experiments, 3 out of 7 cells in a representative example (see their Fig. 3B, C) exhibited repulsive shifts that were followed during recovery by attractive shifts. These ‘rebound’ attractive shifts were of about the same amplitude as the initial repulsive shifts. The time course of these ‘rebound’ shifts is compatible with the time course of adaptation in our experiments. Thus, considering all results the initial effect of adaptation in V1 consists in short-term repulsive shifts; at a secondary stage attractive shifts build up progressively over time. Given its long duration (adaptation and recovery), our protocol is probably more attuned to detect attractive shifts. Another experimental procedure may contribute explaining the differences between our results and previous studies in V1: most groups used a “topping-up” protocol, in which the adapting stimulus is presented as a reminder before each test stimulus. Finally it may be worth noting that Kohn and Movshon [13] failed to induce shifts of preferred orientation in V1, while the same protocol applied in MT induced attractive shifts in monkeys.

Other properties are also influenced by repetitive presentations of one specific feature. For instance cells in area V4 of macaque acquire directional tuning after adaptation, often orthogonal relative to adapting direction [16]. Interestingly, adaptation in MT causes tuning to shift toward the adapted direction [13]. Similar protocol performed on cats' area 17 by measuring spatial frequency tuning instead of orientation produced mostly attractive shifts up to several octaves toward the adapter [17]. Hence, it is remarkable that adaptation changes the tuning of cells depending upon the properties and the area of recording. It thus appears that this short term plasticity is a general property of the mature cortex.

### Mechanisms

Changes in orientation preference through adaptation would be emerging properties of cortical cells. Shifts in tuning curves seem to be regulated by local intra-cortical circuitry involving contiguous orientation domains (shifts rarely exceed 30.0°, see results, Fig. 4). Adapting cells at far flank orientations induced no significant shifts (or rarely). The present investigation reveals that changes of orientation selectivity were set in relation of the adapting duration. Interestingly, we showed that shorter duration of adaptation induced repulsive shifts similarly to what has been reported with topping-up procedure (see above) [11], [13], [19] while longer adaptation time produced attractive displacements of the peak of the tuning curve. Together these results suggest two separate mechanisms for repulsive and attractive shifts. It has been reported that short-term adaptation from seconds to minutes can be the result of a depression of neurons activity at the adapted flank thus leading to apparent repulsive shifts of tuning curves. In general, repulsive shifts were attributed to strengthening of inhibition [4], [11], [34]–[36]. However, solely depression of neuronal responses fails to explain attractive shifts occurring when cells were adapted for a longer period of time. We showed that attractive shifts of the tuning properties mostly emerged from a decline of responses to the original preferred orientation and an increase of discharges for stimuli closer to the adapter (or precisely at the adapter). As adaptation time increases, tuning curves are progressively remodelled: following a few minutes of adaptation suppression near adapted flan override facilitation resulting from repulsive shifts of tuning curves. Then if adaptation is prolonged, suppression becomes limited on the near far flank and facilitation increases toward the adapting stimulus thus revealing attractive shifts (see figure 5). Indeed an alteration in the balance between suppression and facilitation on cells' response could reshape the orientation tuning properties.

We propose that such shifts built-up in time through a process of synaptic reinforcement in the neuronal assembly under constant stimulation by a non-preferred stimulus. At molecular levels, it has recently been proposed that changes in synaptic interactions are occurring via several cellular mechanisms that involved different molecular cascades. At first, changes are mediated via NMDA-glutamate receptors. Then supplementary synaptic strengthening involves metabotropic glutamate (mGlu) receptors. Finally AMPA-glutamate receptors are activated and thereby may reinforce neuronal plasticity [37]. Surprisingly Sabatini et al. [38] reported that it takes approximately 10 minutes after the stimulation begins to stabilize spine growth. Our choice of 12 minutes of adaptation was directed by the fact that in initial experiments shorter time of adaptation produced sometimes less compelling results, whereas after 10 min of adaptation cells displayed more robust changes in orientation preference. Even though both experiments are quite different (*molecular experiments vs. in vivo electrophysiological recordings*), yet this coincidence is rather intriguing. In addition, it has been reported that the time required for recovery of preferred orientation is at least an order of magnitude slower than the time necessary for changing the preferred orientation. Structural changes' occurring in dendritic spines may be responsible for slower recovery processes. Thus, we believe that long-term adaptation leaves traces at the cellular level lasting from several minutes to a few hours. Indeed, we showed that following recovery the second adaptation enhanced cells' responses and reliability at their new acquired preferred stimulus. It is then possible that the first adaptation rests on the NMDA receptors activation, then following the second adaptation the AMPA glutamate receptors become involved leading to further response strengthening.

Our results present clear evidence that orientation-selective responses of adult, hence mature, cortex may change their original preferred orientation selectivity. Presumably the latter arises from hardwired neuronal networks established after the critical period that follows birth. Our data also offer insight into neuronal substrates of perception changes induced by prolonged viewing of single images. In a previous study we carried out the relationship between synchronized activity of neuron spike trains and shifts in orientation preference following adaptation. We demonstrate that the correlated activity between units' action potential become stronger following an adaptation protocol inducing neurons to share closer or even similar orientation preference [20]. Facilitated temporal interactions between groups of neurons may induce a functional advantage that results in strengthening the selectivity of neurons to one particular stimulus (often near the adapter). Hence, adaptation leads to a mutual activation of cells belonging to a common neuronal assembly reinforcing the idea of cellular mechanisms involving local cortical networks. In addition, the increase in cortical responses around the adapting orientation may facilitate the discriminability [5] or perception of oriented contours biased in the direction of the adapting stimulus.

In conclusion our results highlights the malleable nature of basic neuronal properties in adult cortex since repeated adaptations modified both the orientation tuning selectivity and the response strength to the acquired preferred orientation.

## Materials and Methods

### Animal preparation

Twelve adult cats (2.5–3.5 kg) were prepared for electrophysiological recordings from area 17 (superficial layers) as described in a previous report [17]. Experimental procedures followed the regulations of the Canadian Council on Animal Care as well as the US National Institutes of Health guidelines for the care and use of animals in research, and were approved by the Institutional Animal Care and Use Committee of the University of Montreal.

Animals sedated with acepromazine maleate (Atravet, Wyeth-Ayerst, Guelph, ON, Canada; 1 mg·kg^−1^, intramuscular) and atropine sulfate (ATRO-SA, Rafter, Calgary, AB, Canada; 0.04 mg·kg^−1^, intramuscular) were anesthetized with ketamine hydrochloride (Rogarsetic, Pfizer, Kirkland, QC, Canada; 25 mg·kg^−1^, intramuscular). Lidocaine hydrochloride (Xylocaine, AstraZeneca, Mississauga, ON, Canada; 2%) was injected subcutaneously as a local anesthetic during surgery. A tracheotomy was performed for artificial ventilation, and one forelimb vein was cannulated. Animals were then placed in a stereotaxic apparatus. Xylocaine gel (Astra Pharma, Mississauga, ON, Canada; 5%) was applied on the pressure points. For the remaining preparations and recording, paralysis was induced with 40 mg and maintained with 10 mg·kg^−1^·h^−1^ gallamine triethiodide (Flaxedil, Sigma Chemical, St. Louis, MO, USA; intravenous) administered in 5% dextrose lactated Ringer's nutritive solution. General anesthesia was maintained by artificial ventilation with a mixture of N_2_O/O_2_ (70:30) supplemented with 0.5% isoflurane (AErrane, Baxter, Toronto, ON, Canada) for the duration of the experiment. Electroencephalogram, electrocardiogram and expired CO_2_ were monitored continuously to ensure an adequate level of anesthesia. The end-tidal CO_2_ partial pressure was kept constant between 25–30 mmHg. A heated pad was used to maintain a body temperature of 37.5°C. Tribrissen (Schering-Plough, Pointe-Claire, QC, Canada; 30 mg·kg^−1^ per day, subcutaneous) and Duplocillin (Intervet, Withby, ON, Canada; 0.1 mL·kg^−1^, intramuscular) were administered to the animals to prevent bacterial infection. The pupils were dilated with atropine sulfate (Isopto-Atropine, Alcon, Mississauga, ON, Canada; 1%) and the nictitating membranes were retracted with phenylephrine hydrochloride (Mydfrin, Alcon, Mississauga, ON, Canada; 2.5%). Plano contact lenses with artificial pupils (5 mm diameter) were placed on the cat's eyes to prevent the cornea from drying.

A craniotomy (6×6 mm) was performed over the primary visual cortex (area 17/18, Horsley-Clarke coordinates P0–P6; L0–L6). The underlying dura was removed, and once the electrodes were positioned in area 17, the hole was covered with warm agar (3–4% in saline). Melted wax was poured over the agar to provide stability.

### Recording

Multi-unit activity in the visual cortex was recorded by two sets of tungsten microelectrodes (Frederick Haer & Co, Bowdoinham, ME, USA; 10 MΩ at 1 kHz). Each set, consisting of a 4-microelectrode linear array (inter-electrode spacing of 400 µm) enclosed in stainless steel tubing, was controlled by a separate micromanipulator. The signal from the microelectrodes was amplified, band-pass filtered (300 Hz–3 kHz), digitized and recorded with a 0.05 ms temporal resolution (Spike2, and Data wave Technologies Longmont, CO, USA, in initial experiments). Action potentials were sorted out using window discriminator for further off-line analyses. Multi-unit recordings from one electrode usually included 2 (up to 3) well-isolated single units. The spike sorting method was based on cluster classification in reduced space. Z-scores were computed to quantify the difference between clusters. The stability of each cell's activity across conditions was verified qualitatively by visual control of the cluster's disposition and of the waveform's shape. In addition, signal-to-noise ratio was measured as the mean of the waveforms amplitude divided by the noise in the last bin of the temporal window (range: 1.9 to 3.4 ms).

### Visual stimulation

Stimulation was monocular (dominant eye). After clearly detectable activity was obtained, the multi-unit receptive fields (RF) were mapped as the minimum response fields by using a hand-held ophthalmoscope. Eye-screen distance was 57 cm. RF edges were determined by moving a light bar from the periphery toward the center until a response was elicited. These preliminary tests revealed qualitative properties such as dimensions, velocity preference, orientation and directional selectivity. Visual stimuli were generated with a VSG 2/5 graphic board (Cambridge Research Systems, Rochester, England) and displayed on a 21-in. monitor (Sony GDM-F520 Trinitron, Tokyo, Japan) placed 57 cm from the cat's eyes, with 1024×768 pixels, running at 100-Hz frame refresh. Stimuli were sine-wave drifting gratings covering the RF [24]. Contrast was set at 80%. Mean luminance was 40 Cd.m^−2^. Optimal spatial and temporal frequencies were set within the 0.2–0.4 cycles·deg^−1^ and 1.0–2.0 Hz range respectively, where V1 neurons are known to respond well to sine-wave drifting gratings [39], all of the parameters were selected with the aim to evoke maximal firing rates. During experiments, each orientation was presented in blocks of 25 trials lasting 4.1 s with a random inter-trial interval (1.0–3.0 s) during which no stimuli were presented. Orientations were presented in random order. Nine data points (covering 180°; steps of 22.5°) centered on the preferred orientation were selected and used for the rest of the experiment. Peri-stimulus time histograms (PSTH) were recorded. Tuning curves were obtained for moving stimuli, so it is strictly speaking incorrect to describe them as orientation tuning curves. Indeed, orientation is by definition cyclic over the interval 0°–180°, while direction is cyclic over the interval 0°–360° [40]. In other words, for any given orientation, there are 2 possible perpendicular directions for a moving stimulus. Considering that most cells in the cat visual cortex show some degree of direction selectivity [7], [41], a proper description of their responses would rather be a directional tuning curve. However, this distinction will be ignored, as it has been in almost all other studies of orientation tuning in V1.

Once control tuning properties were characterized, an adapting stimulus was presented continuously for 12 minutes on cells' receptive fields (Adaptation I, Fig. 1). The stimulus was a drifting grating whose orientation was generally set 22.5 to 45.0° off the preferred orientations of neurons (contrast, spatial and temporal frequencies were kept at optimal control values, see above). No tests were conducted during this adaptation period. Immediately after adaptation, the orientation tuning curves were determined starting with adapting and control orientations and continuing by recording the remaining stimuli in random order (post-adaptation I tuning curves). Following a recovery period of 60 to 90 min another recording was performed. Then the same adapting protocol was repeated a second time (Adaptation II, Fig. 1) and recordings were achieved a last time. In additional experiments, influence of adaptation length on the orientation preference of cells was determined by increasing the adapter duration from 3 to 12 min (in step of 3 min).

### Data analysis

Tuning curves before and after adaptations were determined by fitting the von Mises function:
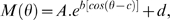
(1)where A is the value of the function at the preferred orientation, c, and b is a width parameter. An additional parameter, d, represents the spontaneous firing rate of the cell [13], [40]. A fit was considered satisfactory if it accounted for at least 80% of the variance in the data. To ensure that recorded cells were properly tuned for orientation, we used an orientation selectivity index (OSI). It was measured using raw tuning curves, by dividing the firing rate at the orthogonal orientations by the firing rate for the preferred orientation, and subtracting the result from one [42], [43]. The closer the OSI is to 1, the stronger the orientation selectivity.

To test the significance of tuning shifts curve fits using von Mises function were generated on cells responses for every trial. As every orientation was applied in a block of 25 presentations, the tuning curve of a given trial represents evoked response for all nine orientations. The above procedure yields 25 tuning curves per experimental condition allowing statistical comparisons between preferred orientations on a trial-by-trial basis (first trial before adaptation against first trial following adaptation, etc…). A paired *t*-test indicated the significance level of shifts [11]. Evoked firing rates of every cell were calculated using the von Mises function applied on the tuning curves of cell and compared across conditions (control, adaptation I, recovery and adaptation II).
